# Curative Effects of Fuzheng Huayu on Liver Fibrosis and Cirrhosis: A Meta-Analysis

**DOI:** 10.1155/2015/125659

**Published:** 2015-06-28

**Authors:** Shu Dong, Qi-Long Chen, Shi-Bing Su

**Affiliations:** Research Center for Traditional Chinese Medicine Complexity System, Shanghai University of Traditional Chinese Medicine, Shanghai 201203, China

## Abstract

The Fuzheng Huayu (FZHY) formula is being used in antiliver fibrosis treatment in China. For systemic evaluation of the curative effects of FZHY on liver fibrosis and cirrhosis progress, a total of 1392 subjects (714 cases and 678 controls) were found to be eligible for meta-analysis in this study. Standard mean differences (SMDs) with 95% confidence interval (CI) were calculated for changes between FZHY groups and controls by employing fixed effects or random effects model. In the overall analysis, alanine transaminase (ALT) (*P* = 0.003, SMD = −0.87, 95% CI: −1.46 to −0.29), total bilirubin (TBil) (*P* = 0.001, SMD = −1.30, 95% CI: −2.10 to −0.50), hyaluronic acid (HA) (*P* = 0.000, SMD = −0.94, 95% CI: −1.30 to −0.58), laminin (LN) (*P* = 0.000, SMD = −0.80, 95% CI: −1.20 to −0.41), type III procollagen (PC-III) (*P* = 0.000, SMD = −1.27, 95% CI: −1.93 to −0.60), and type IV procollagen (IV-C) (*P* = 0.000, SMD = −0.78, 95% CI: −1.05 to −0.51) were decreased after FZHY treatment; however, albumin (ALB) was increased (*P* = 0.037, SMD = 1.10, 95% CI: 0.07 to 2.12) significantly. Furthermore, the Child-Pugh score was reduced significantly and the life quality was improved after FZHY treatment in cirrhosis patients. The results of this meta-analysis indicated that FZHY effectively improves the liver function, alleviates hepatic fibrosis, decreases Child-Pugh score, and relieves TCM symptoms caused by liver dysfunction, indicating that FZHY may contribute to the alleviation of liver fibrosis and cirrhosis.

## 1. Introduction

Liver fibrosis is a consequence of chronic liver disease characterized by replacement of liver tissue by fibrosis, scar tissue, and regenerative nodules, leading to liver dysfunction [1]. Liver fibrosis can progress into liver cirrhosis that causes further hepatocellular dysfunction and increases intrahepatic resistance to blood flow, leading to hepatic insufficiency and portal hypertension, and seriously decreases patients' life quality [2]. Therefore, antiliver fibrosis is very important to prevent the occurrence of liver cirrhosis. However, clinical treatment for liver fibrosis, including early intervention or control of etiologies and hepatic inflammation and regulation of hepatic extracellular matrix (ECM) metabolism and stellate cell (HSC) activation [3, 4], still lacks effective medications.

It has been reported that Chinese medicine significantly contributed to the treatment of liver fibrosis [5–7]. Fuzheng Huayu (FZHY) formula, traditional Chinese medicine (TCM) 319, is a SFDA-approved antifibrotic medicine in China [8]. It consists of 6 Chinese herbal medicines [9] and has functions to promote blood flow, dissolve blood stasis, tonify spirit, and nourish liver [10]. Current studies have showed that FZHY has the effect of antifibrosis in patients with chronic hepatitis B [11, 12] and experimental rats [13] and reduced hyaluronidase and improved the life quality in liver cirrhosis patients [14].

Moreover, FZHY can effectively suppress the pathway related with autocrine activation in HSCs and/or fibrotic liver tissue through inhibiting the vascular endothelial growth factor (VEGF) expression level [13], downregulated *α*-smooth muscle actin (*α*-SMA) expression [15], and regulated the action of transforming growth factor-*β*1 (TGF-*β*1) signaling transduction and Smads activation [16, 17]. FZHY has probably induced the apoptosis of stellate cells by activating p38 mitogen-activated protein kinase (MAPK) and inhibiting stress-activated protein kinases and Jun N-terminal kinase (SAPK/JNK) [18]. In addition, FZHY has also enhanced the activation of hepatic NK cells and the production of interferon-gamma (IFN-*γ*) effectively against liver fibrosis [19].

Furthermore, it has been reported that the antioxidative stress effect of FZHY worked through downregulating the expressions of cytochrome P450 2E1 [20] and tumor necrosis factor receptor type I (TNFR1) [21]. In other diseases, curative effects of FZHY also were being investigated against renal interstitial fibrosis, which is related to the reversal of tubular epithelial-to-mesenchymal transition induced by TGF-*β*1 [10].

Recently, the efficacy of FZHY against liver fibrosis is being evaluated in multicenter, randomized controlled clinical trials. In particular, a phase II clinical trial of FZHY has been successfully completed in USA, which further indicated the effect of FZYH against liver fibrosis [22]. To systematically assess the curative effect of FZHY on liver fibrosis and cirrhosis underlying the clinical trials, a meta-analysis was conducted on all eligible published studies in the current study.

## 2. Methods

### 2.1. Identification and Eligibility of Relevant Studies

**Figure 1:**
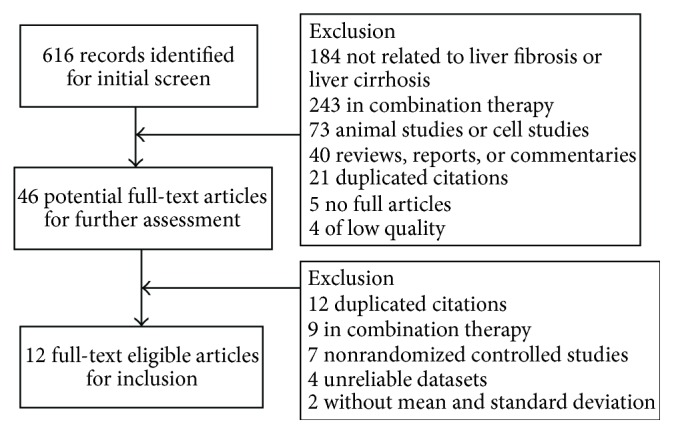
Flow diagram of studies identified with criteria for inclusion and exclusion.

A systematic search was performed using PubMed, Springer, EBSCO Medline, Web of Science, SciVerse ScienceDirect, China National Knowledge Infrastructure (CNKI), and Wan-Fang and VIP database (last search: June 20, 2014) with the following MeSH terms and keywords: Fuzheng Huayu, FZHY, TCM 913, liver fibrosis, liver cirrhosis, and effect. The searches were limited to clinical trials. All eligible studies were examined carefully twice, and their bibliographies were double checked. Twelve full-text eligible articles were selected from 616 candidate studies (Figure 1).

### 2.2. Inclusion Criteria

The following criteria were used for the study selection: (1) evaluating the effects of FZHY as a treatment for liver fibrosis and liver cirrhosis; (2) FZHY alone or based on basic therapy for treatment, other TCM formulas, medication, placebo, or basic therapy for control; (3) full-text articles; (4) random case-control clinical trials; (5) reliable sufficient data estimating mean and standard deviation.

### 2.3. Exclusion Criteria

Studies were excluded as follows: (1) the study did not meet the criteria above; (2) it used FZHY in combination therapy for treatment; (3) it involved animal studies or* in vitro* studies; (4) it did not use the intervention or the outcomes were unreliable; (5) it did not report the primary research such as review articles, letter to the editor, and commentaries or represented duplicate publications of other studies.

### 2.4. Quality Assessment

Methodological quality of RCTs was assessed using five-point Jadad scale [23], including (1) description of randomization; (2) adequate and appropriate randomization method; (3) description of single- or double-blindness; (4) assessors blinded to treatment conditions; (5) description of withdrawals and dropouts. In addition, allocation concealment and dropouts were also taken into the assessment. All trials were reviewed by at least 2 reviewers and any disagreement was resolved through the involvement of a third reviewer in consensus conferences.

The methodological quality of the RCTs included in our study was assessed by the method of Jadad. The scores range from 1 to 5, 1 or 2 being considered as low quality trials and 3 to 5 as high quality.

### 2.5. Data Extraction

Information was carefully extracted from all eligible publications independently by 2 investigators according to the inclusion criteria listed above. For conflicting evaluation, the agreements were reached following consensus and discussion. In each study, the characteristics were collected, such as first author's name, year of publication, design of experiment, general information of participants, history of liver disease, total numbers of trials and controls, interventions for each group, and outcomes of each indicator in trials and controls. We did not define any other indicators in our meta-analysis.

### 2.6. Statistical Methods

The statistical tests were performed with STATA version 11.0 (Stata Corporation, College Station, TX) to quantify and compare the efficacy outcomes of the FZHY group versus controls. Continuous data were reported as mean ± standard deviation (SD). Heterogeneity of studies was checked by the random effects model. *P* value which is equal to or more than the nominal level of 0.05 for the *Q* statistic indicated a lack of heterogeneity across studies, allowing for the use of the fixed effects model, otherwise, perform the random effects model [24]. According to the *P* value of heterogeneity of the results between two groups, we chose the random effects model or fixed effects model to perform data analysis. Continuous outcomes were presented as weighted standard mean differences (SMDs) with 95% confidence intervals (CI). The strength of effect of FZHY on liver fibrosis and cirrhosis was measured by the *P* value of the test of SMD with forest plot.

There are mainly two common tests for publication bias test, Egger's test and Begg's test. In this meta-analysis, the publication bias was diagnosed by Begg's test, which was based on rank correlation method and the value of Pr > |*z*| was calculated to judge if there is publication bias in meta-analysis. The value of Pr > |*z*| less than 0.05 was considered representative of statistically significant publication bias; ≥0.05 was considered representative of no statistically significant publication bias [25].

## 3. Results

### 3.1. Characteristics of Included Studies

According to search strategy defined above, 616 articles were retrieved in this meta-analysis. Based on the inclusion and exclusion criteria, 12 articles [11, 14, 26–35] with 1392 subjects were finally selected (714 cases in treatment group and 678 cases in control group). Male patients with liver cirrhosis were dominated in the subjects. The main characteristics, intervention, and outcome measures of the individual studies were shown in Table 1.

### 3.2. Quality Assessment

The design features clarified as randomization, parallel control, single- or double-blinding, allocation concealment, and withdrawals/dropouts were shown in Table 2. The curative effect assessment performed by the clinicians was not adopted.

In the studies, 12 articles were conducted randomized with a parallel control and 4 articles were with blinding method. The withdrawals/dropouts were also conducted. The measurements of serum indices of liver function and liver fibrosis were independently conducted by laboratory staff in these studies. According to the scores of Jadad scale, 8 studies were of low quality with the score of 2, while other 4 studies were of high quality (Table 2).

### 3.3. Total Effectiveness Rate

The total efficacy rate was assessed in 3 trails [26, 27, 31] among 12 articles. The results showed that the total efficacy rate in FZHY treatment group was no less than 90%, which was stringently higher with controls.

### 3.4. Serum Indices of Liver Function

**Figure 2:**
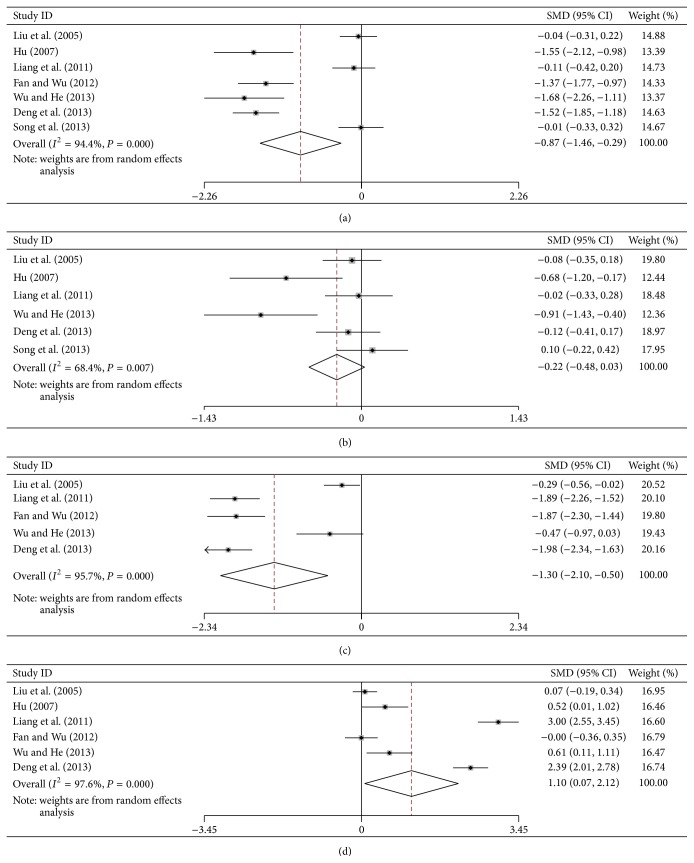
Forest plots of ALT, AST, TBil, and ALB after treatment: (a) ALT, (b) AST, (c) TBil, and (d) ALB.

According to the *P* value of heterogeneity test, a fixed effects model was used for the data analysis before treatment (*P* > 0.05), while a random effects model was used for the data analysis after treatment (*P* ≤ 0.05). The results showed that the values of ALT, AST, and TBil decreased significantly, but the ALB value was increased in FZHY group. Before treatment, the SMDs with 95% CI of ALT, AST, TBil, and ALB were 0.06 (−0.06, 0.19), 0.03 (−0.01, 0.16), −0.07 (−0.22, 0.0.07), and −0.25 (−0.38, −0.11) in patients; however, after treatment, the relative values were changed to −0.87 (−1.46, −0.29), −0.22 (−0.48, 0.03), −1.30 (−2.10, −0.50), and 1.10 (0.07, 2.12), respectively. The forest plots of ALT, AST, TBil, and ALB after treatment were shown in Figure 2 and Supplementary Material (see Supplementary Material available online at http://dx.doi.org/10.1155/2015/125659). The *P* value of SMD was shown in Table 3.

### 3.5. Serum Indices of Liver Fibrosis

**Figure 3:**
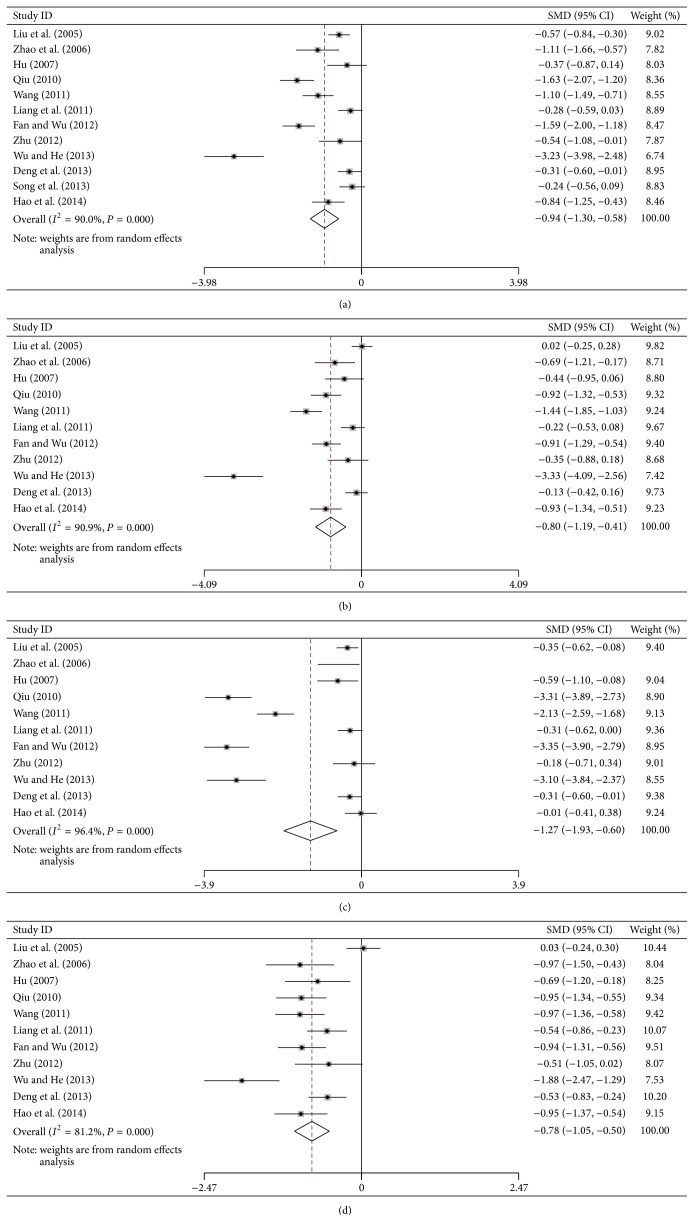
Forest plots of HA, LN, PC-III, and VI-C after treatment: (a) HA, (b) LN, (c) PC-III, and (d) VI-C.

Heterogeneity analyses implied that the data of HA, LN, PC-III, and VI-C before treatment fits a fixed effects model (*P* > 0.05), while the data of HA, LN, PC-III, and VI-C after treatment should use a random effects model (*P* ≤ 0.05). Meta-analysis results showed that the SMDs of HA, LN, PC-III, and VI-C with 95% CI were 0.08 (−0.03, 0.18), 0.01 (−0.10, 0.12), 0.02 (−0.10, 0.13), and 0.11 (−0.00, 0.22) before treatment; similarly, the relative values were −0.94 (−1.30, −0.58), −0.80 (−1.20, −0.41), −1.27 (−1.93, −0.60), and −0.78 (−1.05, −0.51) after treatment. It suggested that the levels of serum HA, LN, PC-III, and VI-C in the treatment groups were lower than those in control groups (Figure 3 and Supplementary Material). The *P* value of SMD was shown in Table 3.

### 3.6. Other Indices

The histologic results showed that liver inflammation grade [11] and fibrosis stage [35] decreased markedly after treatment, whereas no obvious improvement was seen in controls. According to the results of B ultrasound examination [30, 31] and FibroScan [35], FZHY had a better effect on improving the diameter of portal vein and thickness of spleen. In cirrhosis patients [14], FZHY could decrease the Child-Pugh score significantly (*P* < 0.01) compared with the control groups. The effect of FZHY on TCM symptoms [14], life quality [34], and social ability [30] has also been evaluated which showed that FZHY could improve the signs and symptoms of patients compared with the control groups. For the reason that the number of trails evaluating these parameters above was limited, we described these results instead of performing meta-analysis for these data.

### 3.7. Adverse Effect

In this study, 8 trails were observed as adverse events in FZHY treatment. It has been reported that there were one case of mild nausea [33] and 3 cases of mild discomfort in stomach [28]. In another clinical trial, 3 patients in the treatment group experienced mild abdominal distention and 5 in the placebo group developed mild nausea after 2 years of treatment [34]. And the other 4 trails reported no adverse events.

### 3.8. Heterogeneity and Bias Analysis

The heterogeneity of studies was calculated by Cochran's *Q* and *I* ^2^ test, where the *P* ≤ 0.05 or *I* ^2^ ≥ 40% was considered to be significant. The heterogeneity was not significant for these studies (*P* > 0.05) before FZHY treatment; however, the statistical values were stringently heterogeneous in data after treatment (*P* < 0.05) (Table 3).

Furthermore, the publication bias was performed by Begg's test. The forest plots of ALT, AST, TBil, ALB, HA, LN, PC-III, and VI-C were identified, respectively (Figures 2 and 3), and did not reveal any asymmetry for studies. In particular, except for PC-III, the Pr > |*z*| value of other plots was more than 0.05; it indicated that the studies have no publication bias (Table 3).

## 4. Discussion

Liver fibrosis is a common pathologic process for almost all chronic liver diseases which will result in liver cirrhosis or even hepatocellular carcinoma [36]. The primary therapy of antifibrosis in cirrhosis patients is etiological [37] and symptomatic treatment to reduce fibrosis, which has a beneficial impact on portal hypertension and other complications [38]. Currently the main Western medications include interferon and vitamin E [39] which were not approved to be used in clinic officially [40]. Moreover, some Chinese herbal medicines were used against liver fibrosis and cirrhosis such as Danshen and Huangqi injection [41], Qianggan capsule, and Biejia Ruangan tablets [42, 43], but these Chinese herbal medicines have not proved a good and definite effect. Therefore, finding more safe and effective drugs to prevent and reverse liver fibrosis is an urgent task for patients with liver disease.

FZHY formula, as an effective TCM, has been investigated in both animal experiments [44, 45] and clinical trials [46] for several years, which has revealed holistic effects such as improving liver function and serum fibrotic parameters and cirrhosis, decreasing portal pressure, and regulating immune function and amino acids balance [47]. FZHY has also alleviated renal fibrosis [48], against experimental pulmonary fibrosis [49]. Although many multicenter trials were undertaken, considering the limited participants, the coincident trial conditions, and assessment measures, a systemic review is in need to assess the effect of FZHY.

Meta-analysis is systemic and beneficial to assess the accuracy of each trail, which gives a more objective evaluation and explains the heterogeneity between different trails [50]. It has been used in many trails of different diseases [51–53] and used to assess the effect of many kinds of Chinese herbal medicine such as Danshen injection and Huangqi injection against liver cirrhosis [41]. While regarding FZHY, a widely used TCM recipe to treat liver fibrosis and cirrhosis in China, there was no systemic analysis to assess its effects of RCTs recently. In this study, we assessed the curative effects of FZHY on liver fibrosis and cirrhosis. This meta-analysis showed that the curative effect in FZHY-treated groups was better than that of controls. Generally, the serum ALT, AST, TBil, and ALB are the main markers for evaluating liver function; furthermore, the serum HA, LN, PC-III, and IV-C are important markers for evaluating liver fibrosis [54, 55]. In meta-analysis, the forest plots showed that there was significantly statistical difference in SMD test of ALT (*P* = 0.003), TBil (*P* = 0.001), ALB (*P* = 0.037), HA (*P* = 0.000), LN (*P* = 0.000), PC-III (*P* = 0.000), and IV-C (*P* = 0.000) after FZHY treatment, while there was no statistical difference for AST (*P* = 0.084). In 3 trails of liver cirrhosis, the Child-Pugh scores also decreased significantly after treatment (*P* < 0.01) [14]. Histologic results showed that FZHY could decrease liver inflammation grades and fibrosis stage [35], which markedly with the reverse rate 52% in FZHY group in liver biopsy [11].

In addition, clinical research indicated that FZHY also had a better effect on improving the diameter of portal vein and thickness of spleen [30, 31, 35] and effectively reduces the risk of variceal bleeding, improves survival rates in liver cirrhosis patients with varices, especially in the treatment of the capsule and propranolol combination [56], and alleviates ascites [57]. Moreover, FZHY could definitely improve mental disturbance and social activity deficit in patients with chronic hepatitis B-caused cirrhosis [58]. The results of this meta-analysis were also confirmed with self-control clinical trials [59].

TCM syndrome (ZHENG and TCM pattern) determined by clinical symptom and sign can be quantified by TCM syndrome score and used to evaluate the clinical efficacy of Chinese herbal medicine [60]. The effects of FZHY on TCM symptoms [14], life quality [34], and social ability [30] had been evaluated. The results showed that FZHY improves TCM syndrome score and psychology score compared with the control groups. And the accurately TCM pattern differentiation could guide the appropriate TCM treatment with FZHY in patients with hepatitis B-caused cirrhosis [14]. Interestingly, FZHY had also been predicted to have the effect of antihyperlipidemia and antihyperglycemia, through a high-throughput data analysis of hepatitis B-caused cirrhosis treatment [61].

In this meta-analysis, there was no statistically significant heterogeneity in all the comparisons after treatment, and with the outcome of Begg's test, we concluded that there was no statistically significant bias in the overall studies.

Thus, we revealed that FZHY may improve liver function and alleviate liver fibrosis which was consistent with most of the related studies as summarized in meta-analysis. According to the results of Jadad scores, sensitivity analysis, and publication bias test, we believe the outcomes of this meta-analysis were reliable. However, the limitations of the meta-analysis should be acknowledged. Firstly, the methodological design of individual studies was not coincident which included inclusion criteria, source of controls, patients' status, and the drugs used in control group. Secondly, the diversity of treatment dose and the small sample number and the lack of long term follow-ups degraded the validity of the evidence in the clinical trials. Thirdly, liver fibrosis and cirrhosis may be caused by a variety of factors, such as alcohol, medicine, and virus. In this study, the trails included were hepatitis B-caused fibrosis mostly, so this study was limited to reflect the effect of FZHY on liver fibrosis caused by variety of factors. In addition, although some indices of B ultrasound, FibroScan, and the safety evaluation in the trials were reported, they were insufficient to perform meta-analysis; therefore, more high quality random control trails are required. Despite the limitation, our meta-analysis significantly increased the statistical power of the analysis based on substantial number of cases and controls from 12 different studies. The results of meta-analysis did not draw different conclusions, indicating that our results were robust.

## 5. Conclusions

The meta-analysis results suggested that FZHY can effectively improve liver function and life quality by relieved symptoms, alleviating hepatic fibrosis and decreasing Child-Pugh score, with less adverse effect. Therefore, FZHY might be a safe and effective Chinese medicine against liver fibrosis and cirrhosis. Considering that this systematic review had the limitations in the coincident methodological design of each trail and the small number of the included trails, rigorously designed multicenter, double-blind, randomized, and large-scale controlled trials are further required.

## Supplementary Material

According to the P value of heterogeneity test, a fixed effects model was performed for analysis with the data of parameters of liver function (ALT, AST, TBil and ALB) and liver fibrosis (HA, LN, PC-III, and VI-C) before treatment (*P* > 0.05). The results showed that before treatment, the SMDs with 95% CI of ALT, AST, TBil, and ALB were 0.06(−0.06, 0.19), 0.03 (−0.01, 0.16), −0.07 (−0.22, 0.0.07), and−0.25 (−0.38, −0.11) in patients respectively. The forest plots of ALT, AST, TBil, and ALB before treatment were shown in Figure 2. And the SMDs of HA, LN, PC-III, and VI-C with 95% CI before treatment were 0.08 (−0.03, 0.18), 0.01 (−0.10, 0.12), 0.02 (−0.10, 0.13), and 0.11 (−0.00, 0.22). The forest plots of HA, LN, PC-III, and VI-C before treatment were shown in Figure 3.

## Figures and Tables

**Table 1:** Main characteristics, intervention, and outcome measures of the individual studies in the meta-analysis.

Studies and published years [No]	Diseases	Cases (T/C)	Intervention regimens		Time (wk)	Outcomes assessment
			Treatment group	Control group		
Liu et al. 2005 [11]	Hepatitis B-caused fibrosis	110/106	0.93 g per FZHY capsule, five capsules orally, tid	0.93 g per Huoluo Shugan capsule, five capsules orally, tid	24	Histologic examination, liver function, liver fibrosis, B ultrasound examination, safety assessment parameter

Zhao et al. 2006 [26]	Hepatitis B-caused fibrosis	30/30	FZHY recipe orally, twice	Conventional protection liver therapeutics	12	Clinical syndrome, liver fibrosis

Hu 2007 [27]	Cirrhosis	32/30	5 FZHY capsules orally, tid	Conventional protection liver therapeutics	12	Liver function, liver fibrosis

Qiu 2010 [28]	Hepatitis B-caused fibrosis	60/50	0.3 g per tablet in FZHY, 1.5 g tid orally	Conventional protection liver therapeutics	24	Liver fibrosis

Wang 2011 [29]	Hepatitis B-caused fibrosis	60/55	0.3 g per tablet in FZHY, 1.5 g tid orally	Conventional protection liver therapeutics	12	Liver fibrosis

Liang et al. 2011 [30]	Posthepatitic cirrhosis	90/90	4 FZHY tablets orally, tid	Conventional protection liver therapeutics, placebo	24	Social ability score, liver function, liver fibrosis, coagulation, B ultrasound examination, safety assessment

Fan and Wu 2012 [31]	Hepatitis B-caused fibrosis	60/60	0.3 g per tablet in FZHY, 1.5 g orally, tid	Conventional protection liver therapeutics	24	Liver function, liver fibrosis, B ultrasound examination

Zhu 2012 [32]	Posthepatitic fibrosis	28/28	0.3 g per tablet in FZHY, 1.5 g orally, tid	10 g silymarin orally, tid	24	Clinical syndrome, liver fibrosis

Wu and He 2013 [33]	Schistosomiasis hepatic fibrosis	33/31	3 FZHY capsules orally, tid	Conventional protection liver therapeutics	24	Clinical syndrome, liver function, liver fibrosis

Deng et al. 2013 [34]	Posthepatitic cirrhosis	90/90	4 FZHY tablets orally, tid	Four placebo tablets orally, tid	24	Liver function, liver fibrosis, coagulation, hemodynamics, degree of esophagogastric varices, score of symptoms and life quality, adverse events, two-year survival

Song et al. 2013 [14]	Hepatitis B-caused cirrhosis	110/106	0.4 g per tablet in FZHY, 1.6 g orally, tid	0.4 g per tablet in placebo, 1.6 g orally, tid	12	ALT, AST, HA, TCM syndrome score, Child-Pugh

Wang et al. 2014 [35]	Fibrosis	30/30	3 FZHY tablets orally, tid	6 g Anluo Huaxian pill orally, twice	48	Histologic examination, FibroScan, liver fibrosis, B ultrasound examination

No: reference number; T: treatment group; C: control group; wk: weeks.

**Table 2:** The quality evaluation of included trials using Jadad assessment scale.

Studies	Quality					Jadad score
	Randomization	Parallel control	Blindness	Allocation concealment	Withdrawal assessment	
Liu et al. [11]	y	y	y	n	y	4
Zhao et al. [26]	y	y	n	n	n	2
Hu [27]	y	y	n	n	n	2
Qiu [28]	y	y	n	n	n	2
Wang [29]	y	y	n	n	n	2
Liang et al. [30]	y	y	y	n	y	4
Fan and Wu [31]	y	y	n	n	n	2
Zhu [32]	y	y	n	n	n	2
Wu and He [33]	y	y	n	n	n	2
Deng et al. [34]	y	y	y	n	y	4
Song et al. [14]	y	y	y	n	y	4
Hao et al. [35]	y	y	n	n	n	2

y: yes, score = 1; n: no, score = 0.

**Table 3:** SMD, heterogeneity, and publication bias for liver function and fibrosis before and after FZHY treatments.

Parameters	Treatments	SMD (*P* value)	Heterogeneity			Pr⁡>|*z*| (*P* value)
			Chi-squared	*I*-squared	*P* value	
ALT	Before	0.321	7.61	0.212	0.268	0.230
	After	0.003	107.21	0.944	0.000	0.230

AST	Before	0.714	1.78	0.000	0.879	0.452
	After	0.084	15.83	0.684	0.007	0.452

TBil	Before	0.335	5.31	0.247	0.257	0.806
	After	0.001	93.31	0.957	0.000	0.806

ALB	Before	0.001	5.86	0.147	0.320	1.000
	After	0.037	207.03	0.976	0.000	0.452

HA	Before	0.150	1.71	0.000	0.999	0.837
	After	0.000	110.45	0.900	0.000	0.115

LN	Before	0.907	1.63	0.000	0.998	0.640
	After	0.000	109.31	0.909	0.000	0.161

PC-III	Before	0.716	9.25	0.000	0.509	0.043
	After	0.000	275.96	0.964	0.000	0.020

VI-C	Before	0.053	5.00	0.000	0.891	0.533
	After	0.000	53.25	0.812	0.000	0.161
