# Supplementary material for: Curative Effects of Fuzheng Huayu on Liver Fibrosis and Cirrhosis: A Meta-Analysis
Source: Evid Based Complement Alternat Med. 2015 Jun 28;2015:125659. doi: 10.1155/2015/125659 (PMC4499386; doi:10.1155/2015/125659)
Supplement: Supplementary file 1 — According to the P value of heterogeneity test, a fixed effects model was performed for analysis with the data of parameters of liver function (ALT, AST, TBil and ALB) and liver fibrosis (HA, LN, PC-III, and VI-C) before treatment (P > 0.05). The results showed that before treatment, the SMDs with 95% CI of ALT, AST, TBil, and ALB were 0.06(−0.06, 0.19), 0.03 (−0.01, 0.16), −0.07 (−0.22, 0.0.07), and−0.25 (−0.38, −0.11) in patients respectively. The forest plots of ALT, AST, TBil, and ALB before treatment were shown in Figure 2. And the SMDs of HA, LN, PC-III, and VI-C with 95% CI before treatment were 0.08 (−0.03, 0.18), 0.01 (−0.10, 0.12), 0.02 (−0.10, 0.13), and 0.11 (−0.00, 0.22). The forest plots of HA, LN, PC-III, and VI-C before treatment were shown in Figure 3. [file 125659.f1.pdf]

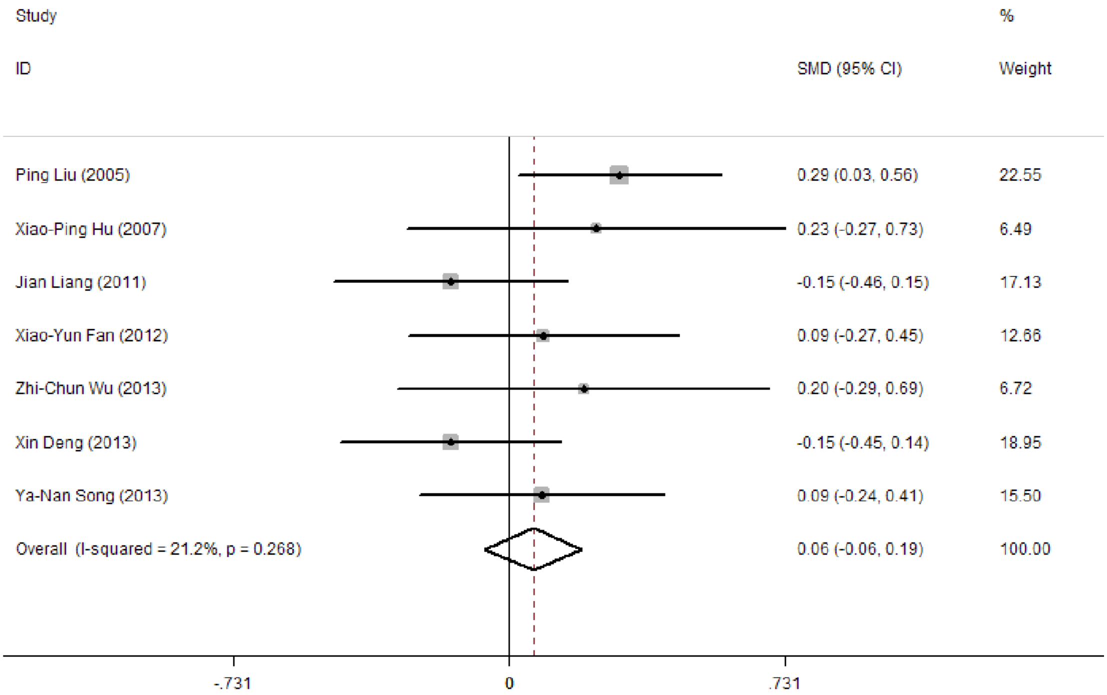
Study
ID
SMD (95% CI)
%
Weight
Ping Liu (2005)
0.29 (0.03, 0.56)
22.55
Xiao-Ping Hu (2007)
0.23 (-0.27, 0.73)
6.49
Jian Liang (2011)
-0.15 (-0.46, 0.15)
17.13
Xiao-Yun Fan (2012)
0.09 (-0.27, 0.45)
12.66
Zhi-Chun Wu (2013)
0.20 (-0.29, 0.69)
6.72
Xin Deng (2013)
-0.15 (-0.45, 0.14)
18.95
Ya-Nan Song (2013)
0.09 (-0.24, 0.41)
15.50
Overall (I-squared = 21.2%, p = 0.268)
0.06 (-0.06, 0.19)
100.00
-.731
0
.731

(e)

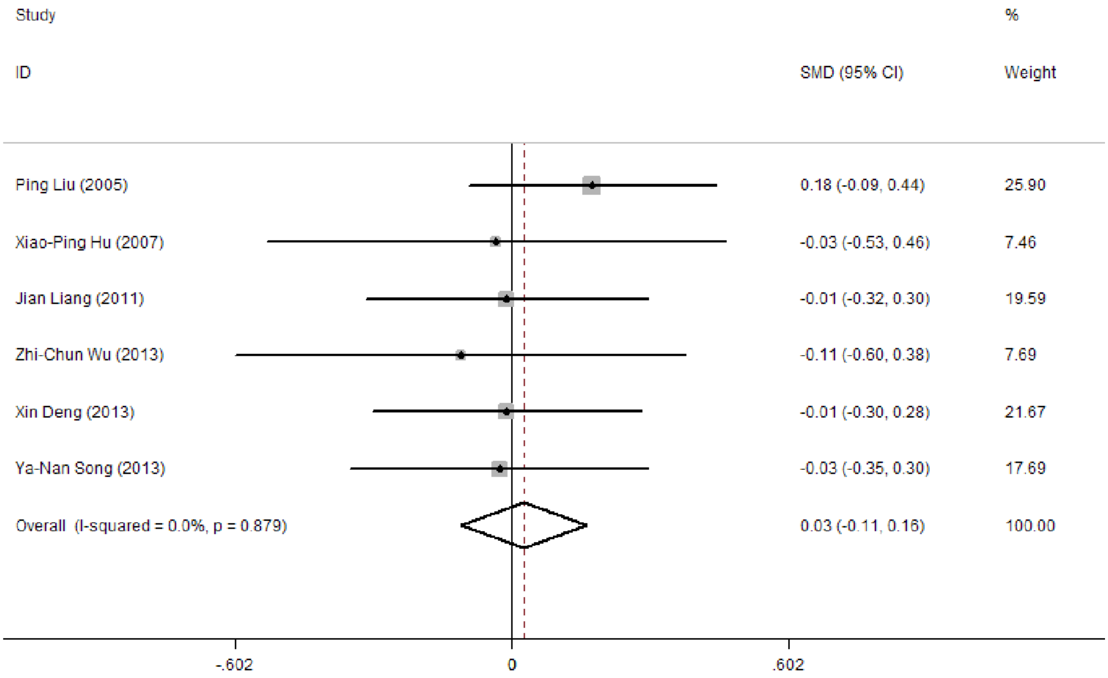
Study
ID
SMD (95% CI)
%
Weight
Ping Liu (2005)
0.18 (-0.09, 0.44)
25.90
Xiao-Ping Hu (2007)
-0.03 (-0.53, 0.46)
7.46
Jian Liang (2011)
-0.01 (-0.32, 0.30)
19.59
Zhi-Chun Wu (2013)
-0.11 (-0.60, 0.38)
7.69
Xin Deng (2013)
-0.01 (-0.30, 0.28)
21.67
Ya-Nan Song (2013)
-0.03 (-0.35, 0.30)
17.69
Overall (I-squared = 0.0%, p = 0.879)
0.03 (-0.11, 0.16)
100.00
-.602
0
.602

(f)

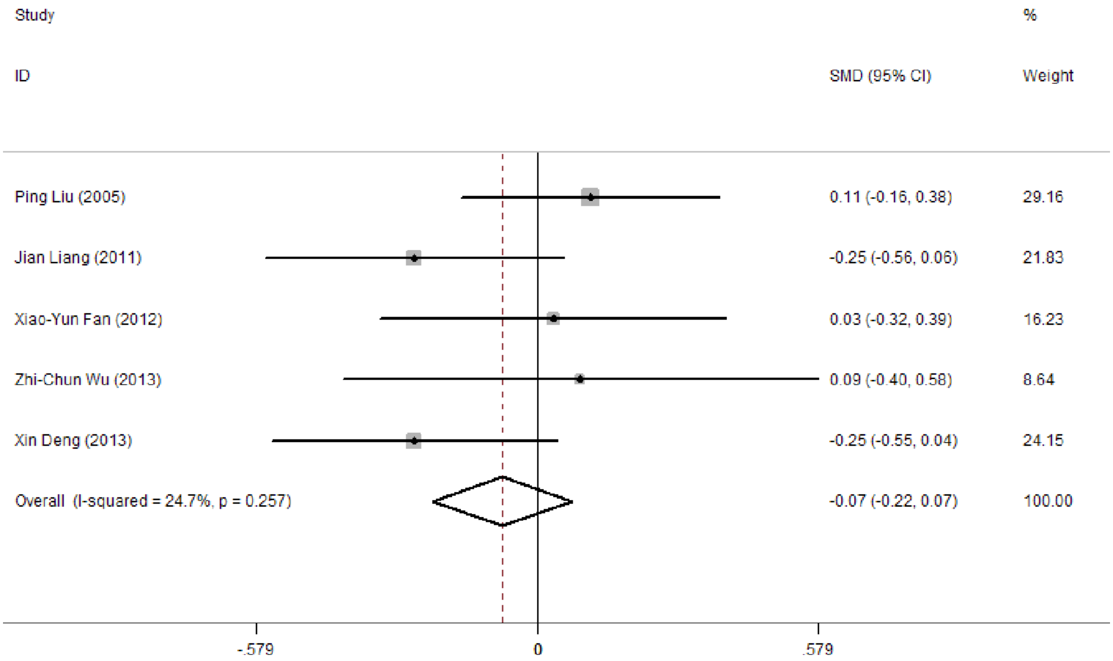

(g)

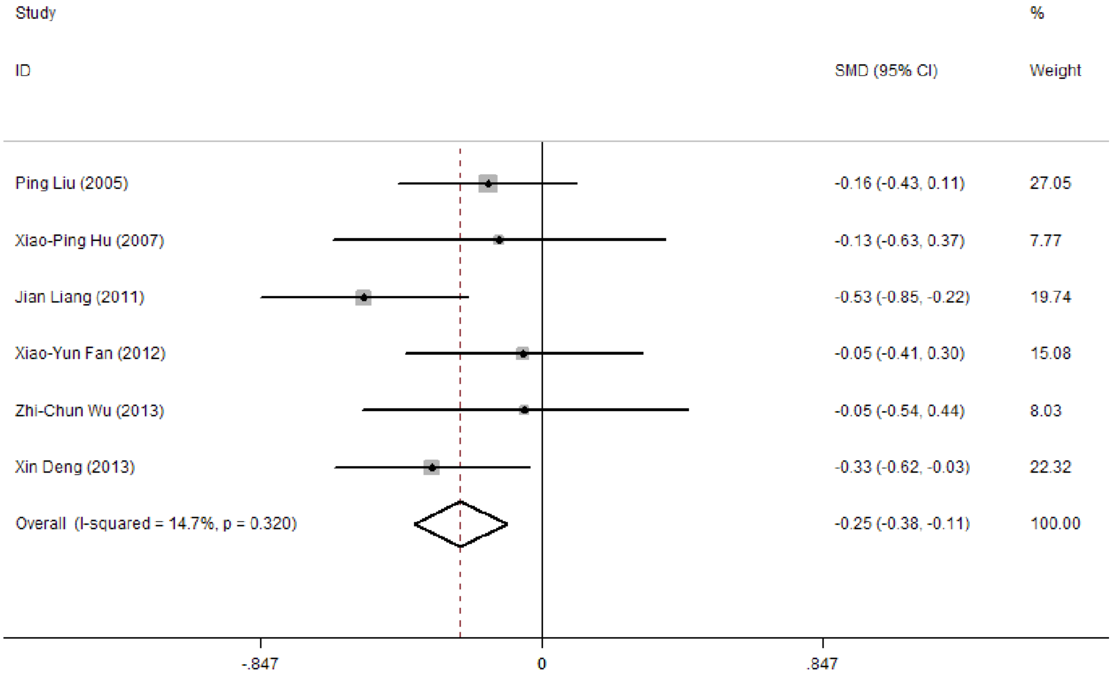

(h)

FIGURE 2: Forest plots of ALT, AST, TBil and ALB before treatment. (e), ALT, (f), AST, (g), TBil, (h), ALB.

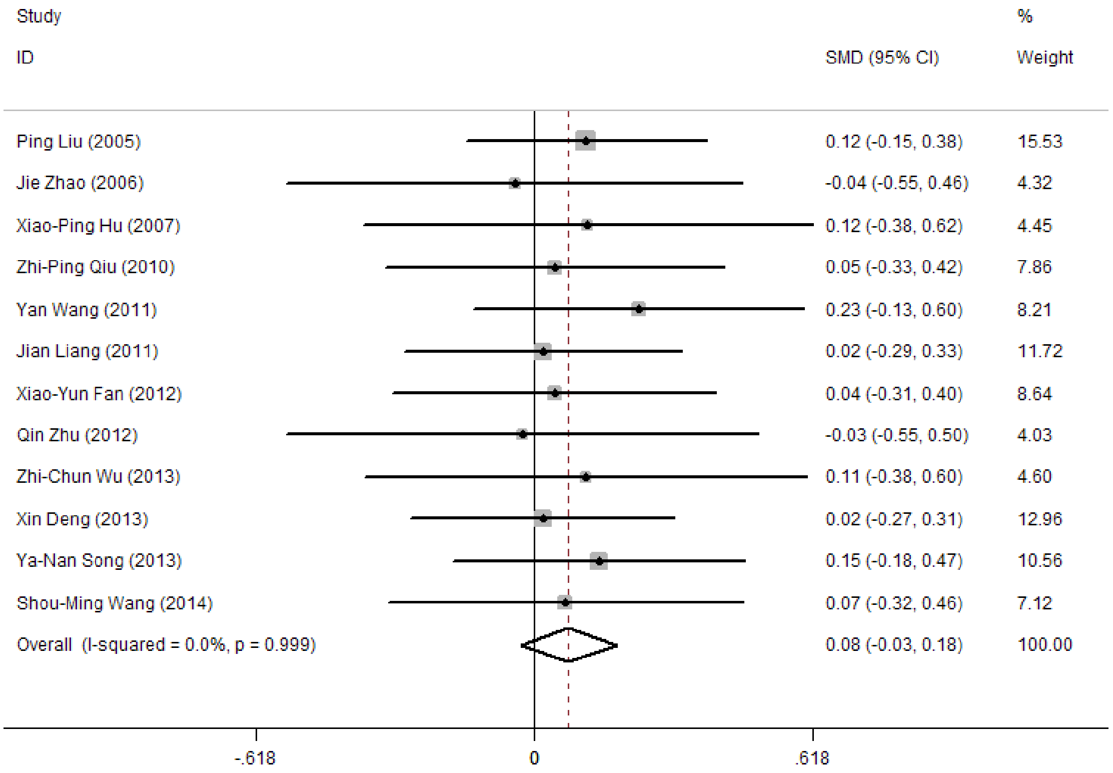
Study
ID
%
SMD (95% CI)
Weight
Ping Liu (2005)
0.12 (-0.15, 0.38)
15.53
Jie Zhao (2006)
-0.04 (-0.55, 0.46)
4.32
Xiao-Ping Hu (2007)
0.12 (-0.38, 0.62)
4.45
Zhi-Ping Qiu (2010)
0.05 (-0.33, 0.42)
7.86
Yan Wang (2011)
0.23 (-0.13, 0.60)
8.21
Jian Liang (2011)
0.02 (-0.29, 0.33)
11.72
Xiao-Yun Fan (2012)
0.04 (-0.31, 0.40)
8.64
Qin Zhu (2012)
-0.03 (-0.55, 0.50)
4.03
Zhi-Chun Wu (2013)
0.11 (-0.38, 0.60)
4.60
Xin Deng (2013)
0.02 (-0.27, 0.31)
12.96
Ya-Nan Song (2013)
0.15 (-0.18, 0.47)
10.56
Shou-Ming Wang (2014)
0.07 (-0.32, 0.46)
7.12
Overall (I-squared = 0.0%, p = 0.999)
0.08 (-0.03, 0.18)
100.00
-.618
0
.618

(e)

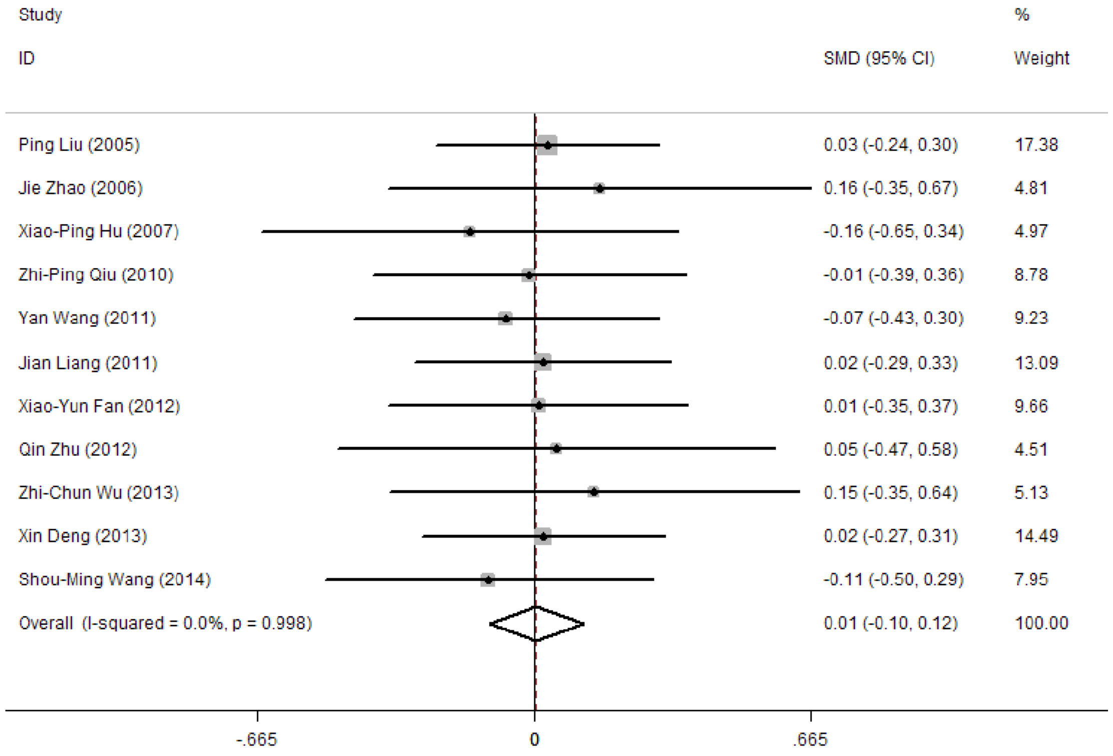
Study
ID
%
SMD (95% CI)
Weight
Ping Liu (2005)
0.03 (-0.24, 0.30)
17.38
Jie Zhao (2006)
0.16 (-0.35, 0.67)
4.81
Xiao-Ping Hu (2007)
-0.16 (-0.65, 0.34)
4.97
Zhi-Ping Qiu (2010)
-0.01 (-0.39, 0.36)
8.78
Yan Wang (2011)
-0.07 (-0.43, 0.30)
9.23
Jian Liang (2011)
0.02 (-0.29, 0.33)
13.09
Xiao-Yun Fan (2012)
0.01 (-0.35, 0.37)
9.66
Qin Zhu (2012)
0.05 (-0.47, 0.58)
4.51
Zhi-Chun Wu (2013)
0.15 (-0.35, 0.64)
5.13
Xin Deng (2013)
0.02 (-0.27, 0.31)
14.49
Shou-Ming Wang (2014)
-0.11 (-0.50, 0.29)
7.95
Overall (I-squared = 0.0%, p = 0.998)
0.01 (-0.10, 0.12)
100.00
-.665
0
.665

(f)

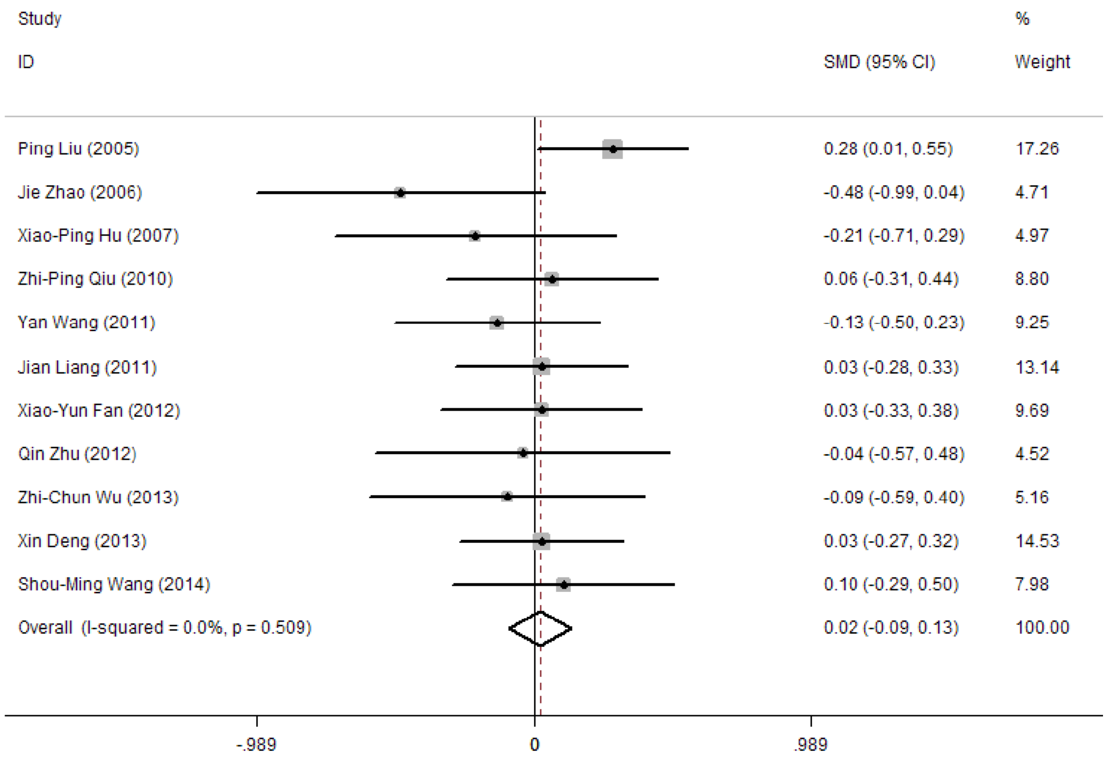

(g)

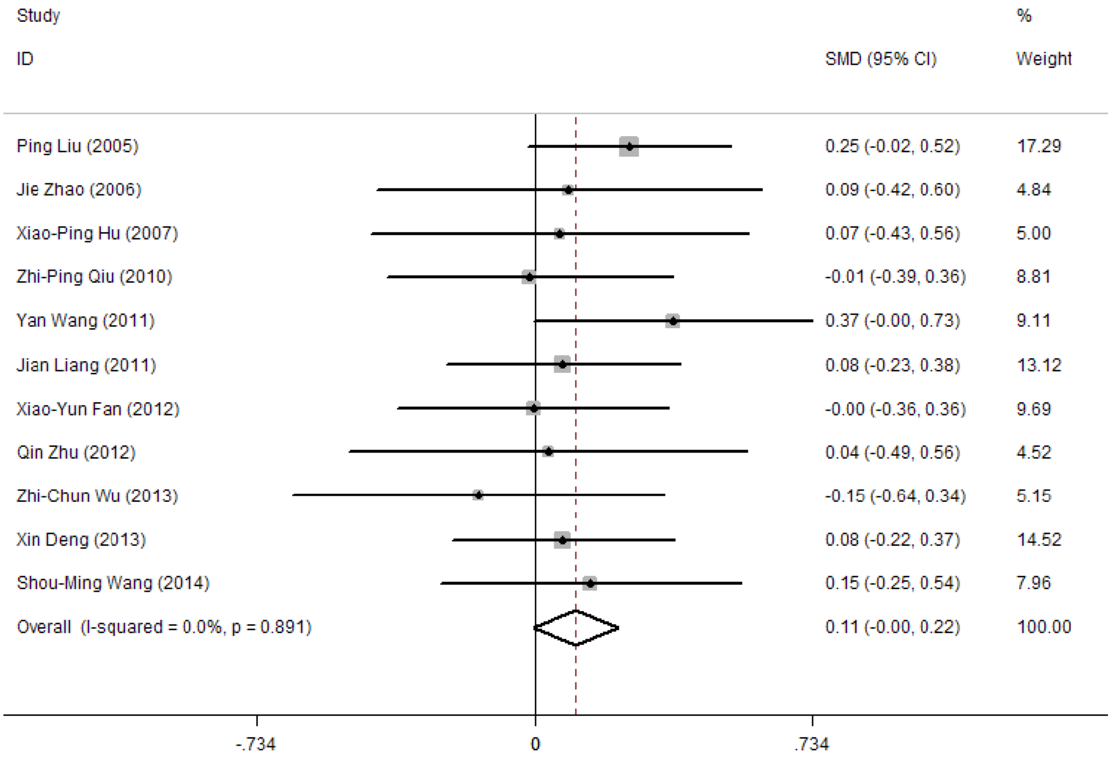

(h)

FIGURE 3: Forest plots of HA, LN, PC-III, VI-C before treatment. (e), HA, (f), LN, (g), PC-III, (h), VI-C.
